# Common cold viruses circulating in children threaten wild chimpanzees through asymptomatic adult carriers

**DOI:** 10.1038/s41598-024-61236-7

**Published:** 2024-05-07

**Authors:** Taylor E. Weary, Tressa Pappas, Patrick Tusiime, Shamilah Tuhaise, Emily Otali, Melissa Emery Thompson, Elizabeth Ross, James E. Gern, Tony L. Goldberg

**Affiliations:** 1 Department of Pathobiological Sciences, University of Wisconsin School of Veterinary Medicine, Madison, WI USA; 2 Department of Paediatrics, University of Wisconsin School of Medicine and Public Health, Madison, WI USA; 3 The Kasiisi Project, Fort Portal, Uganda; 4 Kibale Chimpanzee Project, Fort Portal, Uganda; 5 Department of Anthropology, University of New Mexico, Albuquerque, NM USA

**Keywords:** Respiratory infections, COVID-19, Reverse zoonosis, Uganda, Paediatrics, One Health, Conservation biology, Ecological epidemiology, Viral infection, Paediatric research

## Abstract

Reverse zoonotic respiratory diseases threaten great apes across Sub-Saharan Africa. Studies of wild chimpanzees have identified the causative agents of most respiratory disease outbreaks as “common cold” paediatric human pathogens, but reverse zoonotic transmission pathways have remained unclear. Between May 2019 and August 2021, we conducted a prospective cohort study of 234 children aged 3–11 years in communities bordering Kibale National Park, Uganda, and 30 adults who were forest workers and regularly entered the park. We collected 2047 respiratory symptoms surveys to quantify clinical severity and simultaneously collected 1989 nasopharyngeal swabs approximately monthly for multiplex viral diagnostics. Throughout the course of the study, we also collected 445 faecal samples from 55 wild chimpanzees living nearby in Kibale in social groups that have experienced repeated, and sometimes lethal, epidemics of human-origin respiratory viral disease. We characterized respiratory pathogens in each cohort and examined statistical associations between PCR positivity for detected pathogens and potential risk factors. Children exhibited high incidence rates of respiratory infections, whereas incidence rates in adults were far lower. COVID-19 lockdown in 2020–2021 significantly decreased respiratory disease incidence in both people and chimpanzees. Human respiratory infections peaked in June and September, corresponding to when children returned to school. Rhinovirus, which caused a 2013 outbreak that killed 10% of chimpanzees in a Kibale community, was the most prevalent human pathogen throughout the study and the only pathogen present at each monthly sampling, even during COVID-19 lockdown. Rhinovirus was also most likely to be carried asymptomatically by adults. Although we did not detect human respiratory pathogens in the chimpanzees during the cohort study, we detected human metapneumovirus in two chimpanzees from a February 2023 outbreak that were genetically similar to viruses detected in study participants in 2019. Our data suggest that respiratory pathogens circulate in children and that adults become asymptomatically infected during high-transmission times of year. These asymptomatic adults may then unknowingly carry the pathogens into forest and infect chimpanzees. This conclusion, in turn, implies that intervention strategies based on respiratory symptoms in adults are unlikely to be effective for reducing reverse zoonotic transmission of respiratory viruses to chimpanzees.

## Introduction

Wild great apes are susceptible to serious and often fatal respiratory disease outbreaks. For example, respiratory disease is the leading cause of morbidity and mortality among eastern chimpanzees (*Pan troglodytes schweinfurthii*) at Gombe Stream National Park, Tanzania^1^, and in Kibale National Park, Uganda^2^, two field sites with a continuous research presence since the 1960s and 1980s, respectively. Researchers first documented these outbreaks in 1968, 1975, 1978, and 1987 among Gombe chimpanzees^3,4^. Morbidity ranged from 40–63% and mortality ranged from 8–17%^3^. In 1988, mountain gorillas (*Gorilla beringei beringei*) in Rwanda experienced an outbreak of measles-like respiratory disease that sickened 81% of the group and killed three individuals (10% mortality)^5^. In the intervening decades, outbreaks have continued to occur regularly. Seven of which have sickened nearly all individuals in affected populations, with morbidities of 100% in eastern chimpanzees in Tanzania in 1996^3^, 92–100% in western chimpanzees (*P.t. verus*) in Côte d’Ivoire in 1999, 2004, and 2006^6^, 100% in western chimpanzees in Guinea in 2003^7^, 98% in eastern chimpanzees in Tanzania in 2003^8^, and 92% in mountain gorillas in Rwanda in 2009^9^.

Some outbreaks have caused no mortality, such as among western lowland gorillas (*G. gorilla gorilla*) in the Central African Republic in 2009 (morbidity 50%^10^), mountain gorillas in Rwanda in 2012 (morbidity 60%^11^), and western chimpanzees in Côte d’Ivoire in 2016 (morbidity 27%^12^). However, certain outbreaks caused mortality rates up to nearly half the population, such as among bonobos (*P. paniscus*) in the Democratic Republic of the Congo in 2014 (four deaths; mortality 40%^13^), chimpanzees in Côte d’Ivoire in 1999 and 2009 (six deaths in each outbreak; mortality 19%^6^ and 16%^14^, respectively; eastern chimpanzees in Uganda in 2013 and 2017 (five and 25 deaths; mortality 9%^15^ and 12%^16^, respectively; and chimpanzees in Tanzania in 1993 (11 deaths; mortality 11%^17^. Although respiratory disease outbreaks have thus far been documented only in African great apes, orangutans (*Pongo* spp.) may also be at risk, as evidenced by an outbreak affecting zoo-housed Bornean orangutans (*P. pygmaeus*) in Germany in the 2000s (three deaths; 50% morbidity, 0% mortality)^18^. However, orangutans are less gregarious than African great apes, which may reduce their risk of exchanging pathogens that require close or prolonged contact to be transmitted^19^.

Advances in molecular testing of non-invasively collected samples allowed the etiologic agents of some outbreaks to be identified starting in the mid-2000’s^6,8^. Etiologic agents include viruses from the families *Pneumoviridae,* such as respiratory syncytial virus (RSV^6,10,11,13,14^) and metapneumovirus (MPV^6,8,9,11,16,20^), *Paramyxoviridae* (respirovirus/parainfluenza virus 3, PIV 3^16^), *Picornaviridae* (rhinovirus C, RV-C^15^), *Coronaviridae* (coronavirus OC43, CoV OC43^12^), and *Poxviridae* (Mpox virus, MPXV^21^). With the exception of MPXV, these respiratory viruses share a common epidemiological feature: they circulate commonly in children worldwide and, in aggregate, can be called common cold viruses^22,23^. Common cold viruses generally cause acute, self-limiting infections of the upper respiratory tract, with hallmark symptoms including nasal congestion and discharge, cough, sneezing, sore throat, and lethargy, but in severe cases they can infect the lower respiratory tract or precipitate secondary bacterial infections^23^. Incidence of common colds is highest in children under five years old, especially those frequently interacting with many other children in daycare settings^24^ or in large families^25^. Common cold incidence decreases throughout life^26^; notably, between 57.7% to 97.5% of adult cases are asymptomatic, depending on symptoms definitions^27,28^, and adults who interact with young children are more likely to become infected^25,29^.

Clinical signs for these viruses in great apes resemble those in severely affected children, likely because chimpanzees lack mechanisms of resistance that have evolved in people due to a history of coevolution spanning thousands of years^15,30^. For example, RSV in chimpanzees in Côte d’Ivoire in 2006 was associated with tachypnoea, wheezing, open-mouth breathing, coughing, sneezing, and weakness^6^, all of which are presenting symptoms of RSV pneumonia in severely affected children^31,32^. MPV infection in chimpanzees in Uganda in 2017 caused 12% mortality (see above) with clinical signs including coughing, sneezing, dyspnoea, nasal exudate, and cachexia^16^. One adult female even exhibited persistent wheezing, presumably from emphysema, after the outbreak^16^, a sequela in children who suffer the most severe forms of MPV infections^33^. Two chimpanzees who died from MPV and PIV 3 infections had pericardial effusions^16^, which in humans is a very serious complication from viral infections^34,35^. In contrast, CoV OC43 infection in chimpanzees caused only “sporadic coughing and sneezing”^12^, similar to the mild respiratory symptoms frequently observed in humans infected with common cold coronaviruses^36^. In fatal cases, the proximate cause of death may be secondary bacterial infections caused by *Streptococcus pneumoniae*^1,6,9,13,14,16^, *Streptococcus pyogenes*^1^*, Pasteurella multocida*^6^, or *Klebsiella pneumoniae*^9^. This situation is similar to what is observed in people, where infection with common cold viruses can allow bacterial pathobionts to overgrow^37,38^.

Despite great strides in characterizing the causative agents of respiratory outbreaks in wild great apes, relatively little is known about their transmission pathways into great ape populations from humans (reverse zoonosis, or anthroponosis). However, as mentioned above, many of these viruses share a common trait: they are well known human paediatric viruses that circulate in children. Although a MPXV outbreak in chimpanzees in Côte d’Ivoire in 2017 likely had sylvatic origins^21^, this is the exception to the rule. In general, the risk of reverse zoonotic transmission from humans appears to be greater than the risk from other animals^39,40^. Common cold viruses have no known reservoirs except for humans^41–43^. Epidemiologic modelling indicates that chimpanzee epidemics involving these viruses are point source outbreaks due to rapidly increasing frequencies of new cases over brief periods of time and genetic identity of outbreak-associated virus sequences, rather than the result of prolonged propagation within ape populations^15,16^, suggesting that each resulted from a single reverse zoonotic introduction event followed by rapid transmission within ape social groups^16,44^. Notably, researchers interacting frequently with apes tested positive for the viruses implicated in two outbreaks^10,12^, although in a study of respiratory disease risk factors using long-term field data, increased researcher presence did not to correlate with increased chimpanzee respiratory signs^2^.

It is generally accepted that spending time in close proximity to great apes creates opportunities for reverse zoonotic pathogen transmission via droplets and aerosols^45^ and that humans experiencing active respiratory symptoms pose the highest transmission risk^46^. On that basis, current IUCN guidelines crafted to protect against respiratory pathogens recommend that researchers, tourists, and other people do not enter the forest when they feel ill, they stay at least 10 m away from great apes during encounters, and new visitors should adhere to a 14-day quarantine (increased from seven days in response to the COVID-19 pandemic)^45,47,48^. However, if adults can be infected asymptomatically and shed common cold paediatric viruses unknowingly for weeks^25,49^, such recommendations will likely not be sufficient to prevent transmission. The efficacy of these guidelines is further reduced if people entering the forest are either noncompliant, as has been reported with tourists^50^, or not cognizant of the risks, in the case of park staff uninvolved in ape research or local community members passing through^51^.

Here we describe a prospective cohort study tracking respiratory disease patterns in both humans living in communities surrounding Kibale National Park, Uganda, and wild chimpanzees inside the park from May 2019 to August 2021. We collected respiratory symptoms data and biological specimens (nasopharyngeal swabs from people and faecal samples from chimpanzees), which allowed us to examine statistical associations between various pathogen types and potential demographic and environmental risk factors. Fortuitously, our study took place during the emergence of COVID-19. We were therefore able to examine whether public health measures designed to reduce respiratory disease transmission in people (e.g., closing schools and businesses, closing the international airport, preventing road travel between districts, and increased mask-wearing and handwashing) might affect the frequency of respiratory disease in humans and chimpanzees. This “natural experiment” approach expands on studies performed elsewhere in the world both before^52^ and during COVID-19 emergence^53^. Our findings shed light on patterns of human respiratory disease in western Uganda while also informing evidence-based recommendations to improve chimpanzee health by reducing the risk of reverse zoonosis.

## Results

We collected 1,989 human nasopharyngeal swabs from 264 individuals (538 swabs from 30 adults and 1,451 swabs from 234 children) from May 2019 through August 2021. Over 3,616 total person-months of collection of symptoms data, incidence of respiratory infections was 124.1 cases per 1,000 person-months in children and 64.0 cases per 1,000 person-months in adults. Children were 3.5 times more likely to test positive for a respiratory pathogen than adults (25.8% vs. 9.1% of swabs, X^2^ = 64.1, *p* < 0.0001). RV was by far the most prevalent pathogen identified throughout the study (n = 245 infections, 57.9% of identified pathogens) (Table 1). SARS-CoV-2 and influenza viruses were relatively rare, with only 5 infections (1.2%) and 4 infections (0.9%), respectively. SARS-CoV-2 and influenza B were also the only pathogens for which adults had more infections than children. The bacteria *Legionella pneumophila* and *Mycoplasma pneumoniae*, as well as two types of parainfluenza virus (PIV 1 and PIV 2), were the only pathogens on the RPP not detected in these samples. RV, metapneumovirus (MPV), and parainfluenza virus 3 (PIV 3), the causative agents of three previous outbreaks among Kibale chimpanzees in 2013 and 2017^15,16^, were all identified in our sample set (n = 270 infections, 63.8% of identified pathogens), with high prevalence mostly driven by RV.

**Table 1:** Respiratory pathogens identified in human nasopharyngeal swabs, May 2019–August 2021 (n = 423 out of 1989 swabs, 21.3%).

Pathogen	Adult cases n (%)	Child cases n (%)	Total n (%)
Adenovirus	1 (2.0)	5 (1.3)	6 (1.4)
Bocavirus	0 (0.0)	3 (0.8)	3 (0.7)
Coronavirus (CoV)	7 (14.3)	49 (13.1)	56 (13.2)
CoV 229E	0 (0.0)	3 (0.8)	3 (0.7)
CoV HKU1	0 (0.0)	17 (4.5)	17 (4.1)
CoV NL63	3 (6.1)	26 (7.0)	29 (6.9)
CoV OC43	0 (0.0)	2 (0.5)	2 (0.4)
SARS-CoV-2	4 (8.2)	1 (0.0)	5 (1.2)
Influenza virus	1 (2.0)	3 (0.8)	4 (0.9)
Influenza A (2009 H1N1)	0 (0.0)	3 (0.8)	3 (0.7)
Influenza B	1 (2.0)	0 (0.0)	1 (0.2)
Metapneumovirus (MPV)	0 (0.0)	13 (3.5)	13 (3.1)
Parainfluenza virus (PIV)	2 (4.1)	10 (2.7)	12 (2.8)
PIV 1	0 (0.0)	0 (0.0)	0 (0.0)
PIV 2	0 (0.0)	0 (0.0)	0 (0.0)
PIV 3	0 (0.0)	3 (0.8)	3 (0.7)
PIV 4	2 (4.1)	7 (1.9)	9 (2.1)
Respiratory syncytial virus (RSV)	3 (6.1)	14 (3.7)	17 (4.0)
RSV A	3 (6.1)	3 (0.8)	6 (1.4)
RSV B	0 (0.0)	11 (2.9)	11 (2.6)
Rhinovirus/enterovirus (RV)	35 (71.4)	210 (56.1)	245 (57.9)
*Chlamydophila pneumoniae*	0 (0.0)	67 (17.9)	67 (15.8)
*Legionella pneumophila*	0 (0.0)	0 (0.0)	0 (0.0)
*Mycoplasma pneumoniae*	0 (0.0)	0 (0.0)	0 (0.0)
Total	49	374	423

In addition to age class, respiratory infection risk was driven by calendar month, with significantly increased risk occurring in June (β = 1.197, SE = 0.507, z = 2.363, *p* = 0.0182), September (β = 1.193, SE = 0.513, z = 2.325, *p* = 0.02), and October (β = 1.062, SE = 0.511, z = 2.077, *p* = 0.0378) (Supplementary Table 1). Individual subject identity was the best predictor of respiratory infection risk in each model (GLMM conditional R^2^ for presence/absence of infection = 0.143; LMM conditional R^2^ for symptoms severity = 0.531). Weather variables such as average monthly temperature and humidity were not associated with increased respiratory infection risk (Supplementary Fig. 1).

### Effects of COVID-19 lockdown

**Figure 1:**
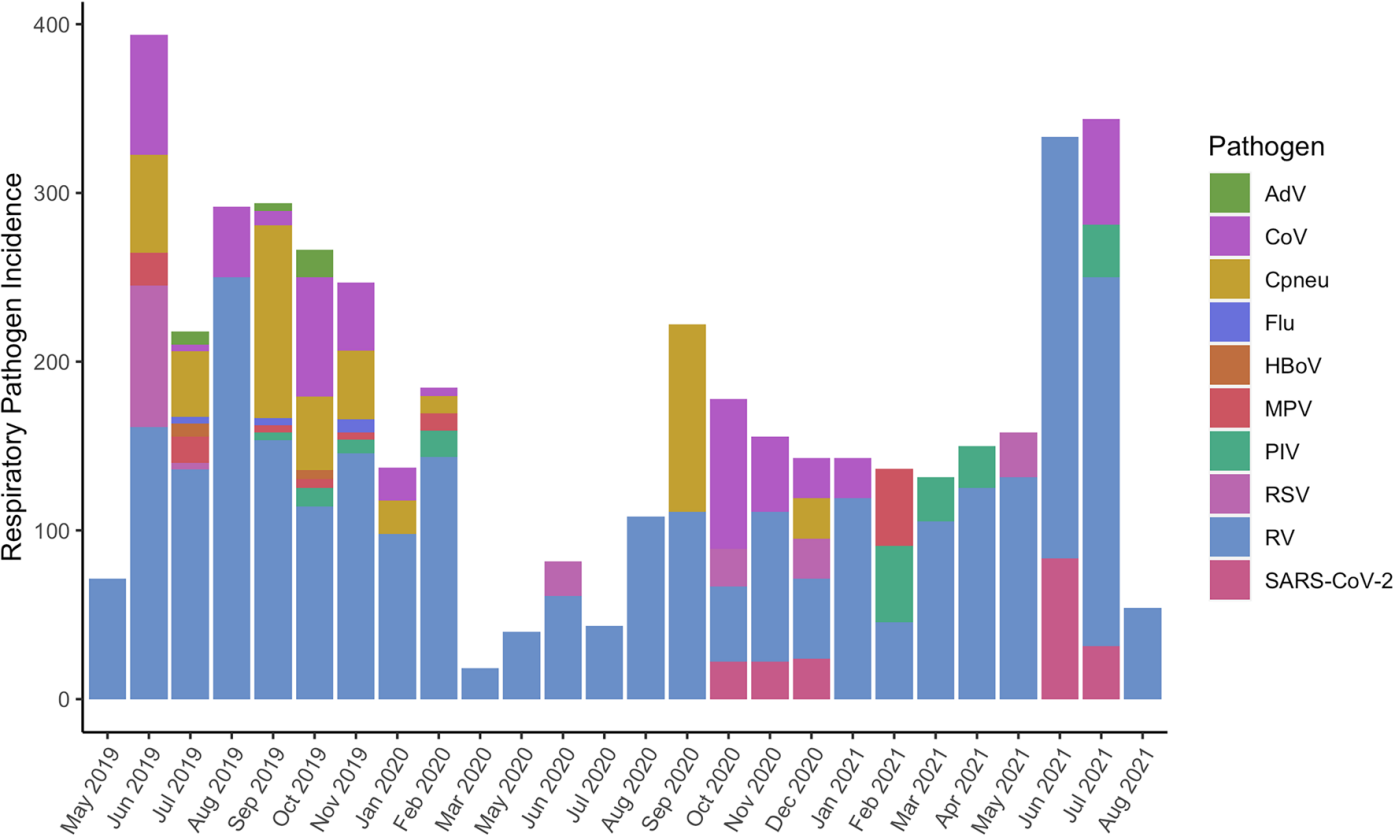
Respiratory pathogen incidence (positive cases per 1000 person-months), May 2019–August 2021. There were no samplings in December 2019 or April 2020. adenovirus (AdV), Chlamydophila pneumoniae (Cpneu), “common cold” coronavirus (CoV 229E, CoV NL63, CoV HKU1, and CoV OC43), influenza virus (Flu), human bocavirus (HBoV), metapneumovirus (MPV), respirovirus/parainfluenza virus (PIV 3 and PIV 4), respiratory syncytial virus (RSV), rhinovirus (RV), and severe acute respiratory syndrome coronavirus 2 (SARS-CoV-2).

Respiratory pathogen incidence decreased during the most stringent part of COVID-19 lockdown compared to the pre-pandemic period (May 2019 to February 2020: 123.1 cases per 1,000 person-months; March to September 2020: 35.5 cases per 1,000 person-months), even when accounting for the decreased number of study participants sampled during lockdown (Kruskal–Wallis test *p* < 0.0001, Dunn’s Test with Bonferroni correction pairwise *p* < 0.0001) (Fig. 1). Respiratory pathogen diversity in the population also decreased significantly after lockdown began (mean pathogens identified per month = 5.6 vs. 2.3, t = 4.0, *p* = 0.0005). After some lockdown measures eased in September 2020 (e.g., reopening of the international airport, students older than 12 years returning to school), incidence and viral diversity increased again, although not significantly (incidence = 92.4 cases per 1,000 person-months: Kruskal–Wallis test *p* < 0.0001, Dunn’s Test with Bonferroni correction pairwise *p* < 0.0001; diversity = 2.7 pathogens identified per month: one-way ANOVA with Tukey HSD *p* = 0.0009, pairwise *p* = 0.30). RV was the most prevalent pathogen each month and the only pathogen present during every monthly sampling (Fig. 1). The first case of SARS-CoV-2 in our data set was identified in a nasal swab collected on 16th October 2020, from an adult.

### Symptoms severity by pathogen

**Figure 2:**
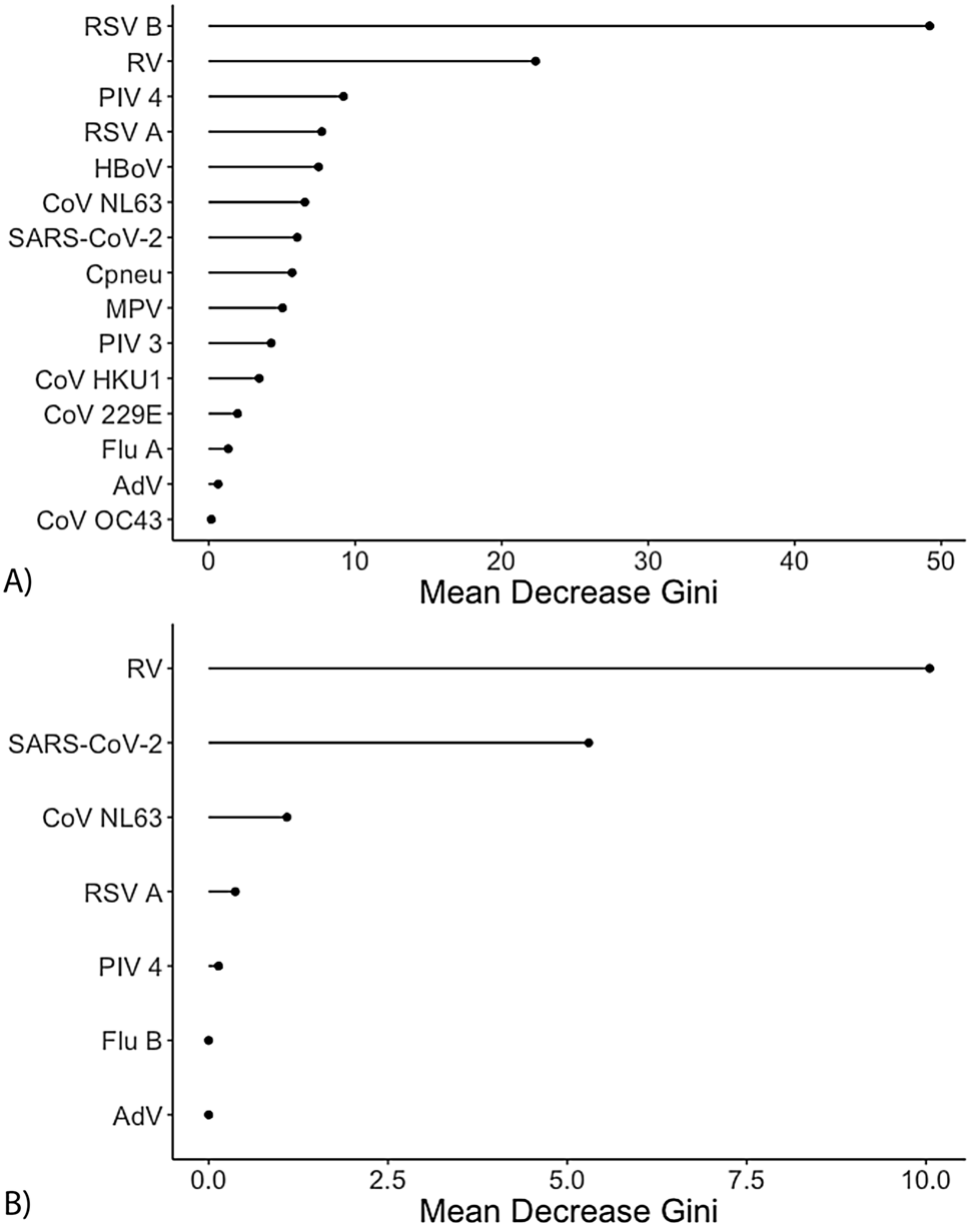
Mean decrease in Gini for each pathogen included in the random forest analysis of symptoms severity by pathogen for (**A**) children (n = 1225) and (**B**) adults (n = 149). Only nasal swabs collected during symptomatic disease were included for analysis. Symptoms severities were quantified with symptoms scores (see Methods). adenovirus (AdV), Chlamydophila pneumoniae (Cpneu), coronavirus (CoV), influenza virus (Flu), human bocavirus (HBoV), metapneumovirus (MPV), respirovirus/parainfluenza virus (PIV), respiratory syncytial virus (RSV), rhinovirus (RV), and severe acute respiratory syndrome coronavirus 2 (SARS-CoV-2).

Random forest analysis indicated that RSV B and RV were the pathogens most important in distinguishing between mild and severe symptoms in children, as determined by symptoms scores (Fig. 2a). Visualisation of the mean decrease in Gini demonstrated that these two viruses each had a substantially larger effect on distinguishing mild from severe disease compared to all other viruses identified in samples collected during symptomatic disease. RSV B in particular was associated with significantly increased symptoms scores (β = 1.752, SE = 0.626, t = 2.800, *p* = 0.0052) (Supplementary Table 2). Among adults, RV and SARS-CoV-2 were the most important in distinguishing between mild and severe symptoms (Fig. 2b), with all other symptomatic pathogens trailing in importance, although the effect of RV or SARS-CoV-2 infection on symptom scores failed to reach statistical significance (Supplementary Table 2).

### Asymptomatic infections

Adults were 5.0 times more likely to have asymptomatic infections than children (67.3% vs. 13.4% of pathogen-positive swabs in cohort, (X^2^ = 80.0, *p* < 0.0001) (Table 2). RV was the pathogen most likely to be carried asymptomatically (67.5% of total asymptomatic cases), both by adults (81.8% of adult asymptomatic cases) and children (58.0% of child asymptomatic cases). All three viruses identified in previous Kibale chimpanzee outbreaks (RV, MPV, and PIV) were present asymptomatically in humans, although MPV was never present asymptomatically in an adult. SARS-CoV-2 was present asymptomatically only once, in an adult.

**Table 2:** Asymptomatic cases (n = 83 out of 423 pathogen-positive swabs, 19.6%).

Pathogen	Adult cases n (%)	Child cases n (%)	Total n (%)
Adenovirus	1 (3.0)	2 (4.0)	3 (3.6)
Coronavirus	2 (6.1)	5 (10.0)	7 (8.4)
CoV HKU1	0 (0.0)	2 (4.0)	2 (2.4)
CoV NL63	1 (3.0)	2 (4.0)	3 (3.6)
CoV OC43	0 (0.0)	1 (2.0)	1 (1.2)
SARS-CoV-2	1 (3.0)	0 (0.0)	1 (1.2)
Metapneumovirus	0 (0.0)	3 (6.0)	3 (3.6)
Parainfluenza virus	1 (3.0)	2 (4.0)	3 (3.6)
PIV 3	0 (0.0)	1 (2.0)	1 (1.2)
PIV 4	1 (3.0)	1 (2.0)	2 (2.4)
Rhinovirus/enterovirus (RV)	27 (81.8)	29 (58.0)	56 (67.5)
RSV	2 (6.1)	0 (0.0)	2 (2.4)
*Chlamydophila pneumoniae*	0 (0.0)	9 (18.0)	9 (10.8)
Total	33	50	83

### Respiratory pathogen co-infections

The vast majority (n = 401, 94.8%) of pathogen-positive swabs tested positive for a single pathogen, but multiple pathogens were detected in the remaining 5.2% (n = 22), indicating presence of respiratory co-infections (Table 3). All but one of these swabs were collected from children. Two pathogens were detected in each swab, except for one swab from a child, from which three pathogens were detected (RV, MPV, and PIV 4). RV was most likely to be present in combination with another pathogen, most often with CoV NL63 (four cases, all in children). SARS-CoV-2 was detected in combination with RV in one swab from a (remarkably) asymptomatic adult. Swabs with one pathogen detected were more likely to be associated with increased symptoms scores than no pathogens detected (β = 0.45, SE = 0.175, t = 2.557, *p* = 0.0106), but co-infections (> 1 pathogen) were not associated with further increased symptoms scores (Supplementary Table 2).

**Table 3:** Respiratory pathogen co-infections (n = 21 child swabs and n = 1 adult swab, n = 22 total).

Co-infecting pathogens	Adult cases	Child cases	Total
RV + CoV NL63	0	4	4
RV + RSV B	0	3	3
MPV + PIV 4	0	2	2
RV + MPV	0	2	2
RV + PIV 4	0	1	1
RV + AdV	0	1	1
RV + HBoV	0	1	1
RV + CoV HKU1	0	1	1
RV + *C. pneumoniae*	0	1	1
RV + MPV + PIV 4	0	1	1
RV + SARS-CoV-2	1	0	1
*C. pneumoniae* + CoV HKU1	0	1	1
*C. pneumoniae* + Flu A	0	1	1
*C. pneumoniae* + RSV B	0	1	1
CoV NL63 + CoV HKU1	0	1	1
CoV NL63 + MPV	0	1	1

### Chimpanzee respiratory disease patterns

**Figure 3:**
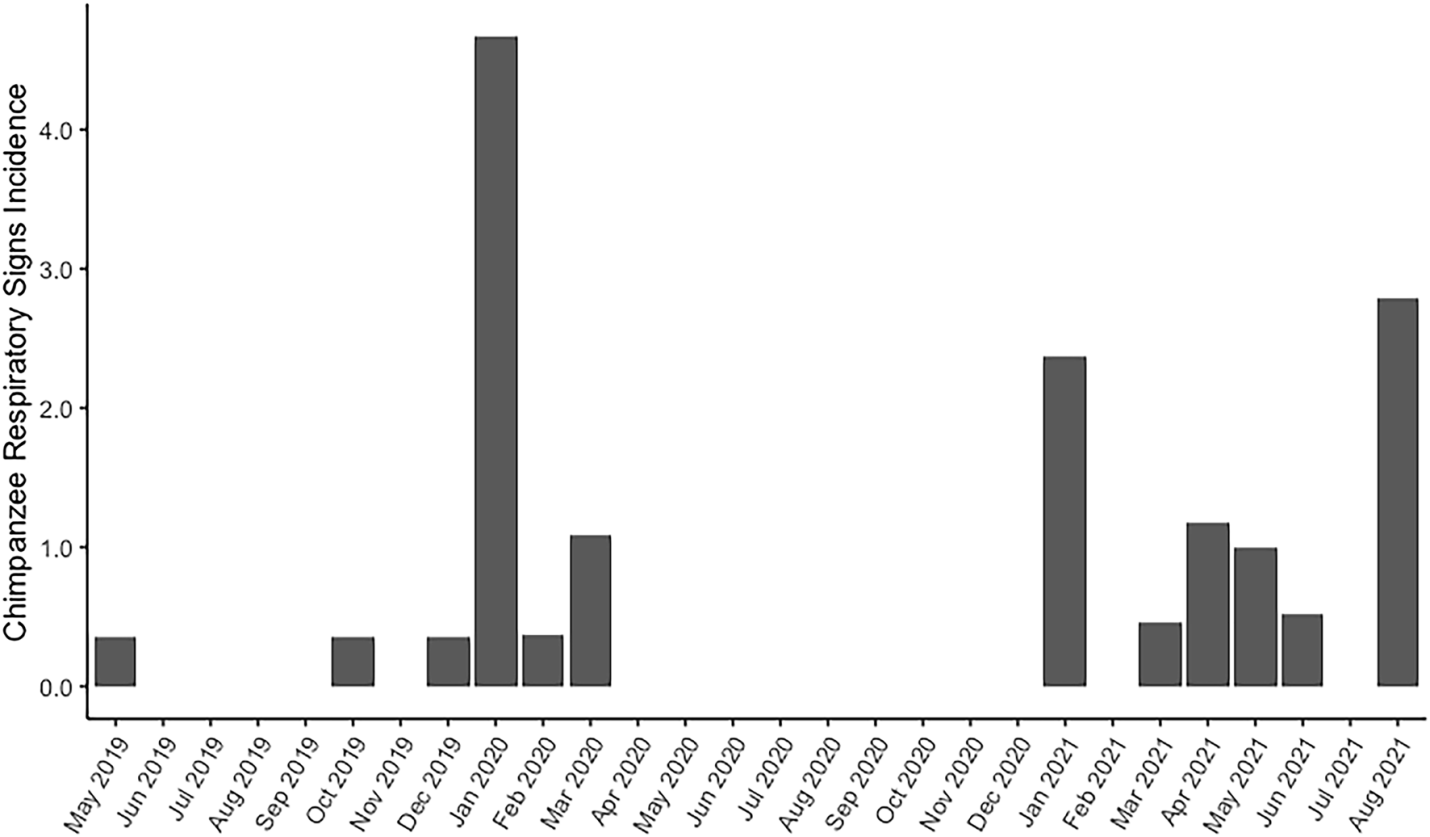
Chimpanzee respiratory disease incidence (cases of respiratory disease per 10,000 chimpanzee observation-hours), May 2019–August 2021.

Throughout the course of the study, we also collected 445 faecal samples from 55 wild chimpanzees living nearby in the Kanyawara social group who have experienced repeated, and sometimes lethal, epidemics of viral respiratory disease, two of which were shown to have originated with humans^15,16^. These chimpanzees experienced no observed respiratory signs between April and December 2020, although the difference of mean incidence rates between this peak lockdown period and before lockdown (May 2019-March 2020) was not significant (t = 1.4, *p* = 0.17) (Fig. 3). The only bout of respiratory disease that met the definition of a community outbreak^2^ during our study occurred in January 2020, before lockdown, with 13 individuals showing clinical signs (e.g., productive cough, nasal discharge, sneezing). Seven chimpanzees tested positive for AdV and six tested positive for non-RV enterovirus (EV) while displaying clinical signs (Supplementary Table 3). Four individuals tested positive for *Streptococcus pneumoniae*, including three juvenile siblings, although two of these individuals also tested positive while healthy before the outbreak in faecal samples from April and October 2019. After some lockdown restrictions eased in late 2020, Kanyawara chimpanzees exhibited a significant increase in respiratory disease incidence in 2021 (April-December 2020 vs. January-August 2021, t = -3.0, *p* = 0.009), although all observed respiratory signs were mild (e.g., unproductive cough or one bout of productive cough). Faecal samples from 2021 were not collected due to research restrictions enacted during that period of the pandemic.

### Chimpanzee metapneumovirus outbreak

**Figure 4:**
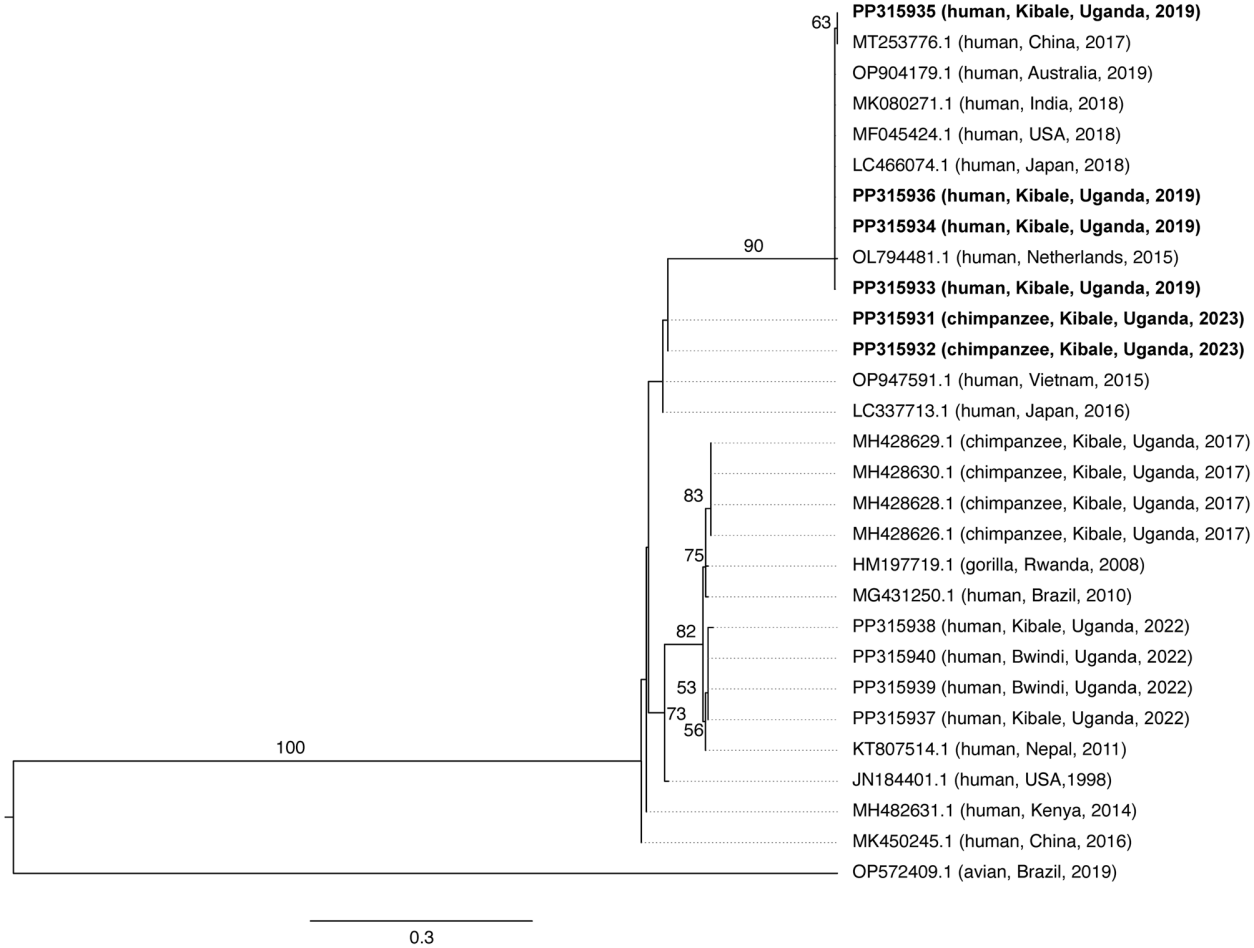
Maximum likelihood phylogenetic tree of metapneumoviruses. The tree is based on a 282-position alignment of fusion protein (F) gene nucleotide sequences and a TN93 + G model of molecular evolution. GenBank accession numbers are followed (in parentheses) by host, location, and year. The tree is outgroup rooted with avian metapneumovirus. Bootstrap values obtained from 1,000 replicates are represented by numbers beside branches; only values ≥ 50% are shown. Scale bar indicates nucleotide substitutions per site.

In February 2023, after we concluded longitudinal sampling of human study participants, the Kanyawara chimpanzees experienced a respiratory disease outbreak that sickened 28% of individuals (n = 18/64) and caused a mortality rate of 4.7% (n = 3/64). Two faecal samples collected from chimpanzees displaying respiratory signs, including a male infant who ultimately died during the outbreak and his mother, tested positive for MPV. The viruses from these chimpanzees were genetically similar to MPV sequences from children in our study collected in 2019 as well as other common MPV sequences circulating globally among people in the previous decade (Fig. 4). Of note, the chimpanzee viruses were genetically distinct from sequences detected during a 2017 MPV outbreak in another chimpanzee community in Kibale National Park^16^ (Fig. 4). Moreover, the average nucleotide distance between the 2023 chimpanzee outbreak sequences and all other sequences in the analysis (4.27 ± 1.17% differences per site) was less than the average nucleotide distance among all sequences in the analysis, including the 2023 chimpanzee outbreak sequences (5.69 ± 1.38% differences per site), indicating that the 2023 chimpanzee-derived sequences nested within the global diversity of MPV sequences analysed. MPV sequences obtained from this study were deposited in GenBank (accession numbers PP315931-PP315936).

## Discussion

In a prospective cohort study of people living near Kibale National Park, Uganda, and chimpanzees living in the park, we found that children were 3.5 times more likely to acquire respiratory infections than adults (124.1 vs. 64.0 cases per 1,000 person-months). RV was the most frequently identified pathogen in people, appearing in every monthly sampling with the highest prevalence (57.9%) of any pathogen. RV and RSV B caused the most severe respiratory symptoms in children, while RV and SARS-CoV-2 caused the most severe symptoms in adults. Paradoxically, RV was also most likely to cause asymptomatic infections (67.5% of total asymptomatic cases), especially in adults (81.8% of adult asymptomatic cases). The highest risk of respiratory pathogen infection in humans occurred in June and September.

Children experienced more frequent respiratory infections than adults, similar to data reported in other studies^25,26,52^. Cumulative lifetime exposures to these paediatric respiratory pathogens lead to enhanced resistance with age due to immunologic memory^54^. This finding concords with studies done around the world showing that these viruses predominantly circulate in children of this age group (3–6 years)^55–57^. Young children also harbour higher viral titres than do older children or adults, which increases opportunities for transmission^58^. Further evidence demonstrates that older siblings^55^ and adults with children at home^25^ are at increased risk for common cold virus infections, with the number of young children in the household positively associated with infection risk^25^. Interestingly, this age-related pattern is inverted in chimpanzees, for whom risk of respiratory illnesses is lowest in juveniles, who, like similarly aged human children, engage most in social play that may put them at increased risk of infectious disease transmission^59^. Instead, respiratory disease risk increases with age in multiple wild chimpanzee populations, likely due to immunosenescence^2^.

The high prevalence of RV supports observations in Uganda^57^ and worldwide^60,61^ that RV is the most important causative agent of the common cold. Mirroring proportions recorded elsewhere^62,63^, RV caused over half (57.9%) of common cold illnesses in our study population. RV was the only pathogen detected during every month of sampling, which may be due to both high RV incidence rates and the fact that RVs are environmentally stable, non-enveloped viruses^64^, meaning that RV can be detected for longer durations by PCR than other common cold viruses^25,49^. Even though many RV infections are mild, RV-C in particular causes severe wheezing illnesses and early-onset asthma in young children^65^. Moreover, the virulence of RV increases with coinfection with other viruses, notably RSV^66^, and RV/RSV coinfection was the second most frequent coinfection pattern observed in our study. High RV and RSV infection rates in early childhood have consequences beyond each individual illness bout. These two viruses are linked to asthma initiation later in childhood and to asthma exacerbations in both children and adults^67^.

Although SARS-CoV-2 cases were rare during our study, 80.0% of such cases (n = 4/5) were in adults. In adults, these infections were more likely to be associated with increased symptoms scores than were other infections. Adult infections were asymptomatic 67.3% of the time, which was five times higher than asymptomatic infections in children. This proportion is similar to that in a U.S. adult population in which 57.7–93.3% had asymptomatic infections^28^. The vast majority of adult asymptomatic infections (81.8%) were RV, mirroring results from the U.S. study^28^. Proposed mechanisms underlying the relative paucity of infection of children with SARS-CoV-2 in our study include activated immune responses from either unrelated infections or recent childhood vaccinations that might protect children from infection^68–70^. Alternatively, adults may have been more susceptible to SARS-CoV-2 due to senescing immune responses, especially in the upper respiratory tract^71,72^.

In addition to age, risk of respiratory pathogen infection varied with calendar month, with significantly increased risk occurring in June and September. Although relative humidity is known to affect respiratory virus stability and therefore transmission rates^73^, weather variables were not associated with increased respiratory infection risk in our dataset; June is during the dry season and September is the monsoon season in Uganda^74^. However, Ugandan school trimesters start at the beginning of February, June, and September each year after monthlong breaks. RV incidence is known to increase at the beginning of school terms in other parts of the world. For example, RV peaks in September in the United States^60^. These peaks generally occur about 11–14 days after the first day of school, when children crowd into classrooms again after vacation^60^. We previously documented that respiratory symptoms increase in February in this study population^75^, although our current data did not reveal a corresponding increase in infections. February is the driest month of the year in Uganda^76^ leading to elevated levels of air pollutants^74,77^. It is therefore possible that nonviral colds synergize with viral infections to contribute to the February peak we found previously in respiratory disease incidence. Future studies of respiratory infection seasonality in Uganda during school years not disrupted by COVID-19 lockdown would be useful for determining whether respiratory infections increase in February in addition to June and September. We note that the nearby Kanyawara chimpanzee community has experienced outbreaks most often in March (i.e., just after February) over the past 20 years^2^. Potential unmeasured variables confounding this complicated causal relationship between young children and chimpanzees through adults entering the forest should be considered in further research. These confounders may include exposure to human pathogens through water sources, trash along the road through the park, or other forest species that interact with humans often, such as baboons^78^.

In combination with previous findings, our results provide evidence that children are an underappreciated source of respiratory viruses that infect chimpanzees. In 2013, a RV-C outbreak sickened between 24 and 40 chimpanzees at a time over the course of a triphasic outbreak and ultimately killed five chimpanzees^15^. MPV and PIV caused simultaneous outbreaks among Kibale chimpanzees in 2017^16^ and, after the current cohort study was completed, MPV caused another Kibale chimpanzee outbreak in 2023. These same pathogens have caused outbreaks among wild apes at other field sites^6,8,9^, as have RSV^6,10,13,14^ and CoV OC43^12^. All these pathogens, generally considered of the highest concern for wild apes, also circulated regularly among children in our study. These children live adjacent to Kibale National Park, and chimpanzees even leave the park to raid crops in the villages where these children and their families live^79^. The parents of these children enter chimpanzee habitats frequently for extractive activities such as firewood and medicinal plant collection as well as infrequent hunting^51,80^, and some of these parents are employed in research that requires them to spend extended periods of time in the forest, sometimes in close proximity to chimpanzees^81^. In aggregate, our data support the hypothesis that asymptomatic adults carry respiratory viruses acquired from local children into the forest and infect chimpanzees, although we acknowledge the evidence from this study is strongest in the inverse (i.e., the absence of infections in children was associated with fewer adult infections and fewer chimpanzee respiratory signs). Experimental investigations of the infectivity of viruses deposited by people on forest fomites (organic or inorganic forest matter, trash, etc.) would be useful to examine this causal relationship further.

The only pathogens detected in chimpanzees during the study period were AdV, EV, and *Streptococcus pneumoniae.* Although AdV and EV have been detected in Cameroonian humans, chimpanzees, and gorillas, reflecting the possibility of pathogen sharing^82^, these viruses have been identified repeatedly in Kibale chimpanzee faecal samples and are not thought to contribute to respiratory disease^15,83^. *Streptococcus pneumoniae* was detected in chimpanzee samples from the January 2020 respiratory disease outbreak at low levels and could be a potential secondary pathogen in chimpanzees weakened by primary viral infections or other circumstances^14^. A limitation of this study is that without chimpanzee faecal samples from 2021 we cannot know for certain that the 2021 cases of respiratory signs were not caused by human pathogens. However, criteria to label these cases a true outbreak^2^ were not met given that all respiratory signs were mild and new cases were sporadic. Although the 2023 MPV outbreak did not occur while we were collecting contemporaneous human samples, it was a fortuitous opportunity to compare viral sequences derived from sick chimpanzees with those derived relatively recently from people living nearby. Indeed, viral sequences obtained from sick chimpanzees were related to those detected from children in our study in 2019 as well as other common globally circulating variants from the previous decade. We acknowledge that there are potential other sources of the outbreak MPV virus, such as ecotourists, national park staff, and other humans entering the park^14^. We emphasize that the pathogens responsible for these outbreaks in apes are ubiquitous in people regardless of occupation or national origin.

Fortuitously, we were able to collect data during COVID-19 emergence. Human respiratory infections decreased significantly during peak lockdown from March to September 2020 (123.1 vs. 35.5 cases per 1,000 person-months) and rebounded as restrictions loosened in late 2020 (92.4 cases per 1,000 person-months). Our finding of reduced respiratory disease incidence during lockdown suggests that public health measures enacted to protect against COVID-19 also reduced transmission of other circulating respiratory pathogens at the same time. Due to the lack of a COVID-19 vaccine early in the pandemic, public health responses relied on nonpharmaceutical interventions (NPIs) such as social distancing, masking, and hand hygiene^53^. NPIs likely disrupted the transmission of SARS-CoV-2 and common cold viruses alike by reducing people’s contact with respiratory droplets, aerosols, and fomites^53^. Our findings mirror those from other parts of the world^53,84–86^.

RV was the only pathogen detected during every month of sampling, including during COVID-19 lockdown when other circulating respiratory viruses all nearly disappeared. This was a global pattern observed during the pandemic^53,84–86^. It is possible that mitigation measures enacted against airborne transmission of SARS-CoV-2 (e.g., social distancing, masking) were less effective against indirect transmission routes more commonly utilized by RV, such as from fomites or contaminated hands^87^. Indeed, there is evidence that masking appears to have lower efficacy against transmission of RV compared to coronaviruses and influenza viruses that rely more on respiratory droplet and aerosol transmission^88^. SARS-CoV-2 and influenza virus infections were in fact conspicuously rare among study participants (1.2% and 0.9% of cases, respectively), perhaps because COVID-19 mitigation measures were more effective against airborne transmission.

Our findings have clear implications for public health. Lockdown was a highly effective strategy for reducing respiratory pathogen transmission among people. Respiratory infections remained lower than pre-pandemic levels after lockdown was lifted. It is possible that health behaviour changes that persisted in these communities after lockdown, such as increased masking and handwashing, continued to prevent disease transmission. We advocate for continuing to promote of respiratory hygiene behaviours, especially among children during times of increased viral activity at the beginning of school terms. Handwashing and decontaminating high-touch surfaces would be especially effective against transmission of RV, which can remain viable on surfaces for days or even weeks^64^, even after desiccation^89^.

Our results can also inform future strategies for great ape conservation. Interventions to reduce respiratory disease in children and heightened biosecurity measures at high-risk times of the school year likely would have the greatest impact in reducing reverse zoonotic transmission risk. Indeed, this idea is encapsulated a “Healthy Children, Healthy Apes” outreach and health promotion program that we have initiated in local communities surrounding the park, based on the research described herein^90^. Reducing respiratory pathogen transmission among young children should reduce the risk of infection in adults who enter great ape habitats. Most of these adult infections are asymptomatic, indicating that biosecurity guidelines that rely on symptoms screening alone may not sufficiently diminish the risk of exposing wild apes to human pathogens. Interestingly, we observed no respiratory signs in the chimpanzee population from April to December 2020, after which cases increased again in 2021, and there was a lethal outbreak in February 2023 after Uganda’s lockdown had been lifted for one year. This finding suggests that reduced respiratory disease incidence in human communities, particularly among children, may have improved health outcomes for chimpanzees living nearby. We acknowledge that this observation could have been coincidental. However, improved respiratory health among wild apes during COVID-19 lockdown has been documented at other field sites^50^, suggesting an association between improved human and great ape respiratory health during the lockdown period. To date, no cases of SARS-CoV-2 have been diagnosed in wild great apes, to our knowledge^50^.

Finally, our results show that health measures designed to protect people can have positive external benefits on animal health, providing an illustration of the popular One Health paradigm^91,92^. In the absence of strict COVID-19 preventive measures, such as lockdown, we recommend enacting programs to reduce disease in preschool- and primary school-aged children living near ape habitats. We also advocate for explicit recognition of the fact that most human viruses that have caused respiratory outbreaks in wild apes are paediatric (i.e., likely to circulate in children who are younger than those who typically interact with apes) and that adults infected with these viruses are usually asymptomatic. Biosecurity measures that adequately account for asymptomatic carriage of these viruses in forest workers, guides, tourists, or other individuals entering ape habitats are most likely to be successful.

## Methods

### Ethical approval

All human biological samples and survey data were collected with institutional approval from Makerere University (2018-077) and the Uganda National Council for Science and Technology (NS 657) under the guiding principles of the World Medical Association Declaration of Helsinki. De-identified data were analysed with institutional approval from the University of Wisconsin-Madison (2019-0229-CR003). All subjects participated voluntarily. Informed consent was obtained from adult participants and parents of child participants (below 18 years old). Assent was obtained from children over eight years old. All informed consent/assent conversations were conducted in study participants’ native language, Rutooro, by native speakers.

The noninvasive procedures used to collect chimpanzee faecal samples in this observational study were approved by the Uganda Wildlife Authority, the Uganda National Council for Science and Technology, and by the Institutional Animal Care and Use Committees (IACUCs) of the University of New Mexico (protocol number 18‐200739‐MC). The study was formally exempt from review by the IACUC of University of Wisconsin-Madison. All procedures complied with the American Society of Primatologists Ethical Principles for the Treatment of Non‐Human Primates.

### Study site, subjects, and sample collection

The design, methods, and study population for the prospective cohort study have been previously reported in detail^75^. Briefly, this study was conducted between May 2019 and August 2021 in Kabarole District in rural Western Uganda. Selection criteria included attending a primary school within 5 km of Kibale National Park for children or working as a research assistant at Makerere University Biological Field Station inside the park for adults. After obtaining written informed consent from adult participants and parents of child participants as well as assent from children over 8 years old, we enrolled 203 schoolchildren (ages 3–6), 30 adults (ages 22–51), and 31 children of those adults (ages 3–11). Each month from May 2019 to February 2020, trained nurses collected monthly nasopharyngeal swabs and respiratory symptoms scores^75^ from all participants. After Uganda instated national lockdown for COVID-19 on March 20, 2020, we obtained permission from study participants and the Ugandan government to continue sampling adult participants and their children with strict biosafety precautions to protect participants and study team personnel. Monthly sampling continued until August 2021, after which sampling was paused until after schools reopened. We conducted a single cross-sectional sampling of respiratory symptoms data in July 2022 to account for changes in respiratory disease frequency that may have occurred following the reopening of schools and the loosening of COVID-19 restrictions on travel and businesses in January 2022^75^.

During the study, trained KCP field assistants collected individual-level observational data on the Kanyawara chimpanzees daily and faecal samples from each individual on a quarterly basis. These chimpanzees are individually identifiable, making such data possible to collect. We compiled these data into measures of clinical signs (coughing or sneezing, further classified as mild or severe), and observation hours per chimpanzee. Even during the strictest lockdown measures (March 2020 through September 2020), a small KCP team was given permission by the Uganda Wildlife Authority to continue entering the park to monitor chimpanzee health. This involved enhanced safety measures, including on-site quarantining of personnel, reduced observer numbers and observation time, increased observation distance, masking, and frequent use of sanitizer. Normal field operations, with continued masking, resumed in October 2020.

We tested human nasopharyngeal swabs and chimpanzee faecal samples, from which respiratory pathogen nucleic acids can be obtained^15,16,93^, using the NxTAG Respiratory Pathogen Panel (RPP) (Luminex Corporation, Austin, TX, USA) as previously described^15,16,94^. Immediately upon collection, both sample types were placed in RNAlater preservation buffer (Thermo Fisher Scientific, Waltham, MA, USA) and stored at − 20 °C until shipment on dry ice to Madison, WI, facilitating molecular analysis.

### Molecular diagnostics

Nucleic acids were extracted as previously described using the NucliSENS EasyMag kit (bioMérieux, Marcy-l’Étoile, France)^15^. The RPP tests for influenza viruses A and B, rhinovirus/enterovirus, adenovirus, parainfluenza viruses 1–4, coronaviruses (CoV NL63, CoV 229E, CoV HKU1, CoV OC43, and SARS-CoV-2), respiratory syncytial viruses A and B, metapneumovirus, human bocavirus, and the bacterial targets *Chlamydophila pneumoniae*, *Mycoplasma pneumoniae*, and *Legionella pneumophilia*. Sensitivity and specificity vary by pathogen but on average are approximately 95% and 99%, respectively^95^. Rhinoviruses were typed by partial sequencing as previously described^95^. The RPP targets include all reverse zoonotic agents identified in chimpanzees to date. Diagnostic testing was performed in the Department of Paediatrics Diagnostic Laboratory of the University of Wisconsin-Madison Hospital and Clinics, following the highest standards of quality control, using FDA-approved protocols as well as positive and negative controls^95^.

In addition to RPP testing, faecal samples from chimpanzees exhibiting respiratory signs from 2019–2021 were also tested for *Streptococcus pneumoniae*, a known secondary respiratory pathogen in chimpanzees^14^. The quantitative PCR (qPCR) assay targeting the *lytA* gene was adapted from methods described previously^96^. PCR reactions were performed in 25 µl volumes consisting of 13.8 µl POWER SYBR Green PCR Master Mix (Thermo Fisher Scientific), 100 µM each primer (forward: 5’-ACGCAATCTAGCAGATGAAGCA-3’; reverse: 5’-TCGTGCGTTTTAATTCCAGCT-3’), PCR-grade water, and 2 µl cDNA. Extracted DNA from *Streptococcus pneumoniae* (Klein) Chester strain 262 (ATCC #49,619, Manassas, VA, USA) and PCR-grade water were used as positive and negative controls, respectively. Thermal cycling parameters consisted of an initial incubation of 50ºC for 2 min and 95ºC for 10 min, followed by 40 cycles of 95ºC for 15 s and 60ºC for 1 min. The qPCR assay was performed using a CFX96 Touch Real-Time PCR Detection System (Bio-Rad, Hercules, CA, USA).

Chimpanzee faecal samples from the February 2023 respiratory disease outbreak and all human samples that tested positive for MPV by RPP were confirmed by single-plex MPV PCR and Sanger sequencing as described previously^97^. Reverse transcription was performed using random hexamer primers and the High Capacity cDNA Reverse Transcription Kit (Thermo Fisher Scientific) with 10 µl each RNA and master mix. Amplicons were 410 bp in length.

### Phylogenetic analyses

To infer phylogenetic relationships, we aligned F gene fragments (282 bp) from all available MPV sequences in GenBank with MUSCLE^98^ to generate a maximum likelihood tree using PhyML 3.0^99^ and SMS (TN93 + G model of molecular evolution)^100^ with 1,000 bootstrap replicates. We displayed the resulting tree using FigTree 1.4.4^101^. We used MEGA 11^102^ to calculate genetic distances (nucleotide *p*-distance ± standard error, 1,000 bootstrap replicates) of MPV sequences within and between the 2023 chimpanzee outbreak virus and closely related MPVs.

### Inferential statistics

To examine associations between respiratory infections, their severities, and putative risk factors, we used linear mixed models (LMMs) and generalized linear mixed models (GLMMs) using the lmerTest package in R^103^. The response variables for the LMMs and binomial GLMMs were symptoms scores and presence/absence of respiratory infection, respectively. Parametric model assumptions were assessed with Shapiro–Wilk tests for verification of normality and with Levene's test for verification of homogeneity of variances. Model diagnostics were performed using the lmerTest package in R. We included subject ID as a random effect to control for multiple sampling of individuals. Significance of full models and of random effects was evaluated using log-likelihood ratio tests. The best models were selected using Akaike’s information criteria (AIC)^104^. All statistical tests were performed at α = 0.05 (two-sided).

To avoid model overparameterization, we ranked the importance of model variables (either individual pathogens or respiratory disease risk factors) in distinguishing between presence and absence of infection (Supplementary Fig. 1) or mild and severe respiratory disease (Fig. 2; Supplementary Fig. 2) using random forest classification, a machine learning tool that generates a large number of decision trees based on random, independent sampling of a data set to make predictions about and classify those data^105^. We used the randomForest package in R^106^ to model these relationships in our human study participants. We computed the models using the function *randomForest*, with settings ntree = 50,000 and mtry = 5, followed by the importance function to rank the relative contribution of each pathogen/risk factor in distinguishing between severe and mild symptoms or presence/absence of respiratory infection via the mean decrease in Gini values, an index of node impurity (i.e., how effectively the forest classifies samples).

Average monthly weather data for 1991–2021 available via the European Centre for Medium-Range Weather Forecasts (ECMWF) for Fort Portal, Uganda, included rainfall (mm), rainy days per month, hours of sunlight per day, temperature (°C, including maximum, minimum, and average values), and humidity (%)^76^.

### Supplementary Information

Supplementary Information.

## Data Availability

Sequence data that support the findings of this study have been deposited in GenBank with the Accession Numbers PP315931-PP315936.
