# Author Correction: Common cold viruses circulating in children threaten wild chimpanzees through asymptomatic adult carriers

**DOI:** 10.1038/s41598-024-67589-3

**Published:** 2024-07-17

**Authors:** Taylor E. Weary, Tressa Pappas, Patrick Tusiime, Shamilah Tuhaise, Emily Otali, Melissa Emery Thompson, Elizabeth Ross, James E. Gern, Tony L. Goldberg

**Affiliations:** 1 Department of Pathobiological Sciences, University of Wisconsin School of Veterinary Medicine, Madison, WI USA; 2 Department of Paediatrics, University of Wisconsin School of Medicine and Public Health, Madison, WI USA; 3 The Kasiisi Project, Fort Portal, Uganda; 4 Kibale Chimpanzee Project, Fort Portal, Uganda; 5 Department of Anthropology, University of New Mexico, Albuquerque, NM USA

Correction to: *Scientific Reports* 10.1038/s41598-024-61236-7, published online 07 May 2024

The Funding section in the original version of this Article was omitted.

The Funding section now reads: “This work was supported by the Morris Animal Foundation (D21ZO-044 to TLG) and the U.S. National Institute on Aging and NIH Office for Research on Women’s Health (R01-AG049395 and R37AG049395 to MET and TLG), with additional support through a Dr. Gregory D. Bossart Memorial One Health Scholarship funded by the Georgia Aquarium and administered by the One Health Commission (to TEW), a Graduate Student Research Award funded by the University of Wisconsin Global Health Institute (to TEW), the Disney Conservation Fund (to ER), and the ARCUS Foundation (G-PGM-2011-3337 to ER).”

The original Article has been corrected.

