# Supplementary material for: Common cold viruses circulating in children threaten wild chimpanzees through asymptomatic adult carriers
Source: Sci Rep. 2024 May 7;14:10431. doi: 10.1038/s41598-024-61236-7 (PMC11076286; doi:10.1038/s41598-024-61236-7)
Supplement: Supplementary file 1 — Supplementary Information. [file 41598_2024_61236_MOESM1_ESM.pdf]

Supplementary Information

Supplementary Figure 1. Mean decrease Gini for each variable included in the random forest analysis of respiratory infection risk by risk factor for adults and children (n=1,989 nasal swabs).

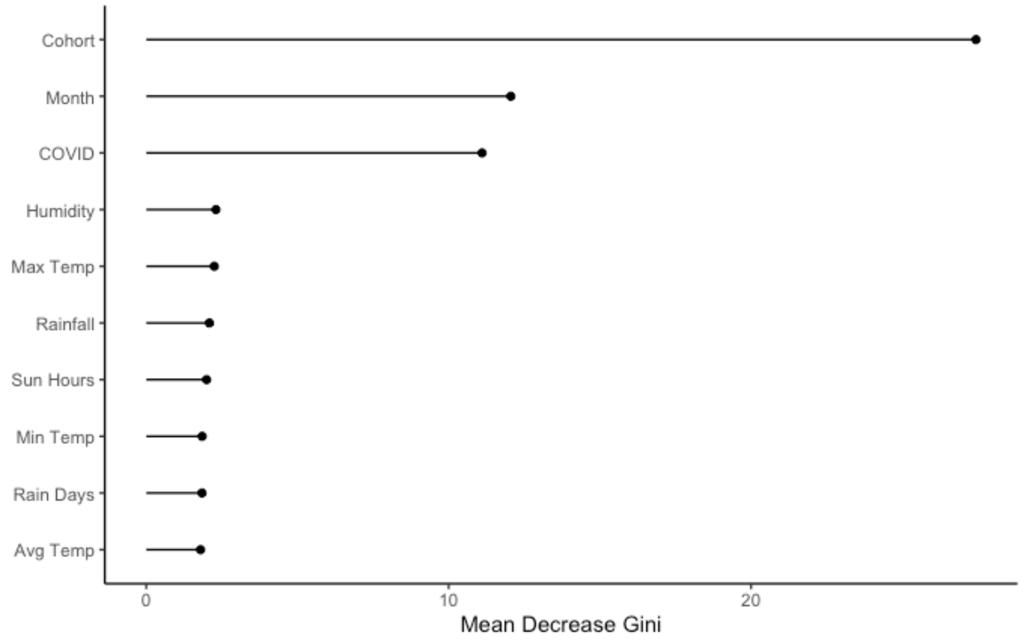

Supplementary Figure 2. Mean decrease Gini for each variable included in the random forest analysis of severity of symptoms (symptoms scores) by risk factor for adults and children (n=1,989 nasal swabs).

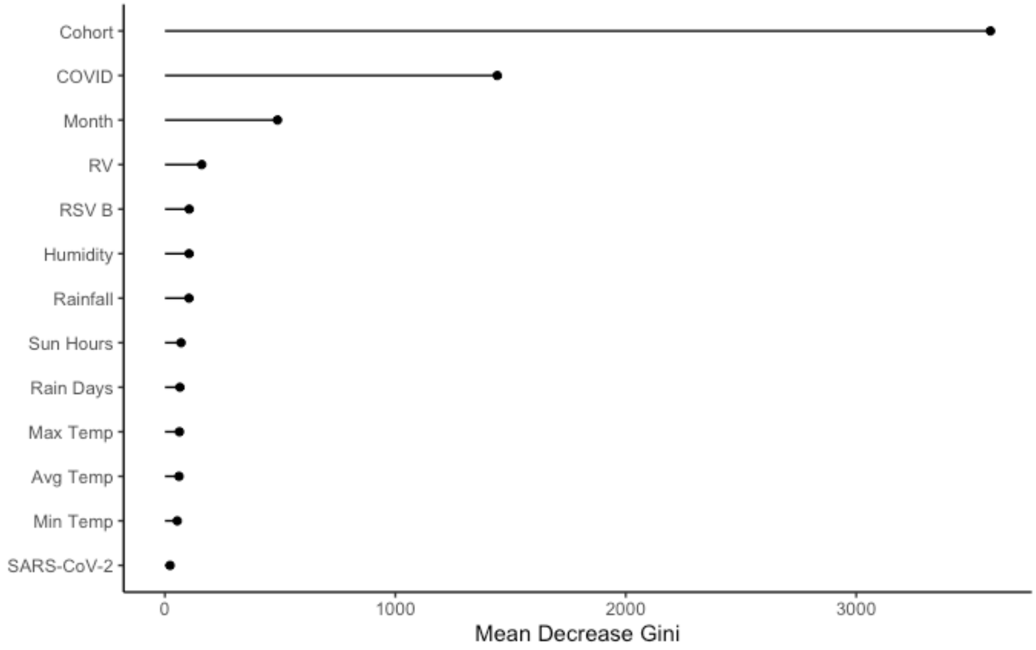

Supplementary Table 1. Results of binomial generalized linear mixed model (GLMM) for effects of age class, COVID-19 lockdown, and calendar month on presence/absence of respiratory pathogen infection. Statistically significant effects ($p<0.05$) are bolded.

**Fixed effects[a]**	**β**	**SE**	**Wald X^2 test**	**p**
(intercept)	-3.0087	0.5247	-5.734	<0.0001
**Forest workers' children[b]**	**0.7753**	**0.2243**	**3.457**	**0.0005**
**Schoolchildren[b]**	**1.0557**	**0.2281**	**4.628**	**<0.0001**
COVID-19 lockdown	-0.1770	0.2190	-0.808	0.4190
Month[c]:	-	-	-	-
January	0.8289	0.5651	1.467	0.1424
February	0.4842	0.5231	0.926	0.3546
April	1.0795	0.6474	1.668	0.0954
May	0.5805	0.5725	1.014	0.3106
**June**	**1.1967**	**0.5065**	**2.363**	**0.0182**
July	0.8283	0.5043	1.642	0.1005
August	0.6145	0.5560	1.105	0.2691
**September**	**1.1928**	**0.5131**	**2.325**	**0.0201**
**October**	**1.0618**	**0.5112**	**2.077**	**0.0378**
November	0.8564	0.5084	1.684	0.0921
December	0.9111	0.6439	1.415	0.1571
**Random effect**	**Variance**	**SD**	**Likelihood ratio test (X^2)**	**p**
Subject	-1.3664	0.0753	-18.15	<0.0001

[a]The marginal and conditional R^2 of the GLMM were 0.102 and 0.143, respectively.
[b]Reference category for cohort: adult forest workers.
[c]Reference category for month: March, the month with the lowest risk of respiratory infection (see Figure 1).

Supplementary Table 2. Results of linear mixed model (LMM) for effects of age class, pathogen richness, and specific viral infections on severity of symptoms (symptoms scores). Statistically significant effects ($p<0.05$) are bolded.

**Fixed effects[a]**	**β**	**SE**	**95% CI**	**df**	**t**	**p**
(intercept)	0.7563	0.2415	[0.301, 1.233]	153.7	3.132	0.0021
**Forest workers' children[b]**	**1.3368**	**0.3402**	**[0.707, 2.037]**	**157.4**	**3.929**	**0.0001**
**Schoolchildren[b]**	**3.5711**	**0.2643**	**[3.053, 4.046]**	**167.8**	**13.509**	**<0.0001**
**Richness: 1 pathogen[c]**	**0.4466**	**0.1747**	**[0.101, 0.795]**	**1813.9**	**2.557**	**0.0106**
Richness: 2+ pathogens[c]	0.7418	0.4798	[-0.134, 1.717]	1844.7	1.546	0.1222
**RSV B**	**1.7520**	**0.6257**	**[0.592, 3.010]**	**1775.3**	**2.800**	**0.0052**
RV	-0.205	0.2079	[-0.615, 0.191]	1808.2	-0.988	0.3232
SARS-CoV-2	0.5924	0.8753	[-1.114, 2.342]	1670.6	0.677	0.4986

[a]The marginal and conditional R^2 of the LMM were 0.336 and 0.531, respectively.
[b]Reference category for cohort: adult forest workers.
[c]Reference category for richness: zero pathogens.

Supplementary Table 3. Pathogens detected in paired fecal samples from Kanyawara chimpanzees before and during quarters with observed respiratory clinical signs (n=32 samples from 14 individuals).

ID	Respiratory Signs	Quarter	Month	Year	AdV	EV	Spn
AT		Q4	NOV	2019			
AT	Y	Q1	JAN	2020			
BB		Q3	SEPT	2019	■		
BB	Y	Q4	DEC	2019			■
DL		Q2	MAY	2019			
DL	Y	Q1	FEB	2020			
ES		Q4	DEC	2019			
ES	Y	Q1	JAN	2020			
ES		Q2	MAY	2019			
ES	Y	Q3	AUG	2019			
JU		Q4	NOV	2019	■		
JU	Y	Q1	MAR	2020		■	
MX		Q4	OCT	2019	■	■	
MX	Y	Q1	MAR	2020	■		
OL		Q2	APR	2019	■		
OL	Y	Q2	MAY	2019			■
OL		Q4	OCT	2019	■	■	
OL	Y	Q1	JAN	2020			
OM		Q4	OCT	2019		■	■
OM	Y	Q1	MAR	2020	■		■
OTB		Q2	APR	2019			■
OTB	Y	Q1	MAR	2020	■		
PB		Q4	NOV	2019	■	■	
PB	Y	Q1	JAN	2020			
QK		Q4	OCT	2019			
QK	Y	Q1	FEB	2020			
QT		Q4	OCT	2019	■		
QT	Y	Q1	JAN	2020			
QV		Q4	OCT	2019	■		
QV	Y	Q1	JAN	2020			
UN		Q4	OCT	2019			
UN	Y	Q1	JAN	2020		■	

Pathogens are adenovirus (AdV), non-rhinovirus enterovirus (EV), and *Streptococcus pneumoniae* (Spn). Black cells indicate positive results for the individuals listed by two-letter abbreviation in the first column. All EV-positive samples were typed to rule out rhinovirus.
